# Advances in modeling cellular mechanical perceptions and responses via the membrane-cytoskeleton-nucleus machinery^☆^

**DOI:** 10.1016/j.mbm.2024.100040

**Published:** 2024-01-26

**Authors:** Hongyuan Zhu, Run Miao, Jin Wang, Min Lin

**Affiliations:** a The Key Laboratory of Biomedical Information Engineering of Ministry of Education, School of Life Science and Technology, Xi'an Jiaotong University, Xi'an 710049, PR China; b Bioinspired Engineering and Biomechanics Center (BEBC), Xi'an Jiaotong University, Xi'an 710049, PR China

**Keywords:** Cellular mechanobiology, Mechanical models, Mechanosensitive receptors, Cytoskeleton, Nucleus

## Abstract

Mechanical models offer a quantitative framework for understanding scientific problems, predicting novel phenomena, and guiding experimental designs. Over the past few decades, the emerging field of cellular mechanobiology has greatly benefited from the substantial contributions of new theoretical tools grounded in mechanical models. Within the expansive realm of mechanobiology, the investigation of how cells sense and respond to their microenvironment has become a prominent research focus. There is a growing acknowledgment that cells mechanically interact with their external surroundings through an integrated machinery encompassing the cell membrane, cytoskeleton, and nucleus. This review provides a comprehensive overview of mechanical models addressing three pivotal components crucial for force transmission within cells, extending from mechanosensitive receptors on the cell membrane to the actomyosin cytoskeleton and ultimately to the nucleus. We present the existing numerical relationships that form the basis for understanding the structures, mechanical properties, and functions of these components. Additionally, we underscore the significance of developing mechanical models in advancing cellular mechanobiology and propose potential directions for the evolution of these models.

## 1 Introductions

The mechanical interactions between the cell membrane, cytoskeleton, and nucleus play a crucial role in the cellular mechanosensing and response [1,2]. The cell membrane acts as a barrier between the cell and its external environment, and the mechanosensitive receptors on the membrane serve as key points for various force-dependent cellular activities [3]. These receptors not only anchor the cell to the extracellular matrix or neighboring cells but also initiate signaling pathways in response to mechanical cues [4]. The cytoskeleton, composed of actin filaments, microtubules, and intermediate filaments, serves as the primary mechanical structure within the cell [5]. It provides structural stability and integrity to the cell, allows for cell shape changes and motility, and participates in force transmission throughout the cell [[6], [7], [8]]. The nucleus, as the central hub for genetic and metabolic activities, is also involved in mechanosensing and response. Mechanosensitive components within the nucleus, such as lamin proteins [9], nuclear pores [10], and chromatin [11], allow the nucleus to detect and respond to mechanical forces. Nuclear deformation in response to external mechanical stimuli can affect gene expression and signaling pathways [12,13]. Overall, the interconnected components of the cell membrane, cytoskeleton, and nucleus form a unified machinery for mechanosensing and response. Understanding these mechanical interactions is crucial for unraveling the complex mechanisms underlying cellular mechanobiology.

While experimental studies have provided valuable insights into the mechanobiology of the membrane-cytoskeleton-nucleus axis, they often have limitations in terms of cost, technical complexity, and spatiotemporal resolution [14,15]. These limitations make it challenging to obtain a comprehensive understanding of the mechanical interactions and subsequent mechanotransductions within cells. Mechanical models, on the other hand, offer conceptual frameworks that can integrate scattered experimental data across different scales of space and time [16]. By mathematically representing the mechanical behaviors of the membrane, cytoskeleton, and nucleus, these models can provide a holistic view and a systematic understanding of the complex phenomena involved in the force transmission axis [17]. Moreover, mechanical models can guide experimental design by providing valuable insights and testable hypotheses. They can predict the outcomes of experiments and identify key variables or parameters that need to be measured. This helps researchers optimize their experimental approaches and focus on the most relevant aspects of cellular mechanobiology. The iterative relationship between mechanical models and experimental findings is essential for deepening our understanding of cell mechanics. As new experimental data emerges, mechanical models can be refined and updated to better capture the underlying mechanisms. In turn, improved models can generate new hypotheses and guide further experiments, leading to a continuous cycle of knowledge advancement. Therefore, the integration of mechanical models and experimental studies is crucial for advancing the field of cellular mechanobiology and gaining deeper insights into the mechanical interactions within cells.

**Fig. 1:**
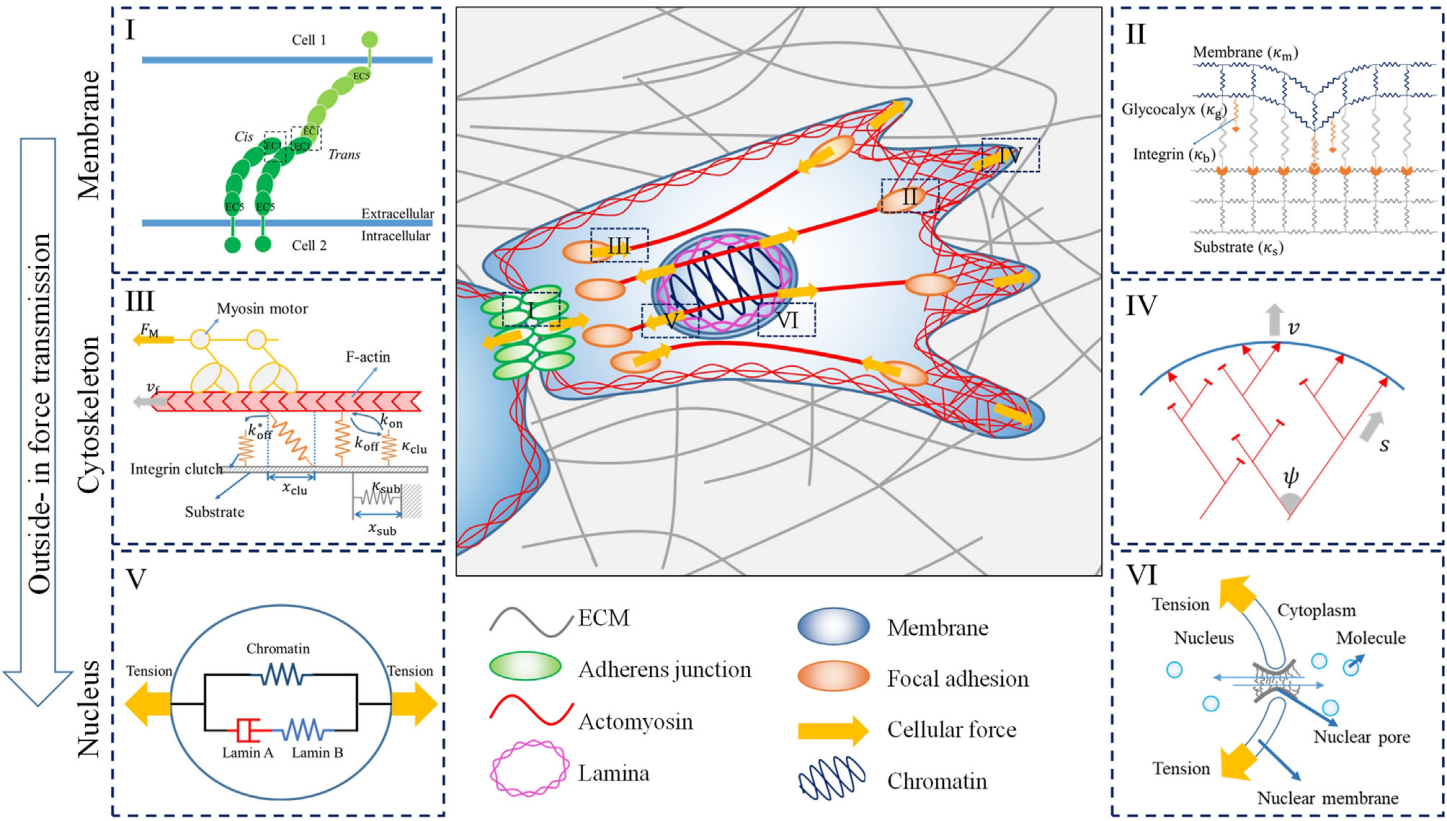
Modeling outside-in force transmission of a motile cell through the membrane-cytoskeleton-nucleus machinery. The yellow arrows showed the direction of forces provided by the cell. The dashed boxes marked with Roman numerals denote the typical structures around membrane, cytoskeleton and nucleus respectively. The snapshots marked with the same Roman numerals on both sides are the mathematical views of the corresponding structures. (**I, II**) Schematics of typical mechanical models of adhesions mediated by mechanosensitive receptors on cell membrane, cadherin (I) and integrin (II) respectively. (**III, IV**) Schematics of typical mechanical models of actomyosin cytoskeleton structures, filopodia (III) and lamellipodia (IV) respectively. (**V, VI**) Schematics of typical mechanical models of nuclear mechanical property (V) and nuclear mechanotransduction (VI) respectively. (I-VI) are adopted from references [50,83,105,111,117,169] respectively.

In this review, we introduce the mechanical models describing how mechanics and biology collaborate to drive the membrane-cytoskeleton-nucleus machinery in the subcellular scale (Fig. 1). We summarize the structure-property-function relationships within the three main mechanical components in cell respectively, with emphasis on how mechanical models evolve to give clearer, more general pictures for these mechanics-driven cellular behaviors. We also address the strengths and limitations of these mechanical models. Finally, we give a perspective on open challenges in understanding the mechanical cues in cell physiology. By reviewing the current state of mathematical modeling in the context of the membrane-cytoskeleton-nucleus machinery, we hope to shed light on the importance of these models in unraveling the complex mechanisms underlying cellular mechanobiology and provide insights for future research directions.

## 2 Models for dynamics of mechanosensitive receptors

Mechanosensitive receptors (*e.g.* cadherins [3,18,19], integrins [15,20,21], ion channels [[22], [23], [24]], immunoreceptors [25,26]) play a pivotal role in mechanosensing on the cell membrane. These receptors execute their functions through a series of stepwise activities, including activation, binding, and clustering. To describe the molecular activities of these membrane receptors, two simulation methods have proven particularly effective: molecular dynamics (MD) and Monte Carlo (MC) models. MD models utilize solved crystal structures of proteins and employ simulations based on Newton's laws to predict the molecular structures and mechanical interactions of proteins [[27], [28], [29], [30]]. Consequently, MD models offer precise and detailed descriptions of nanoscale structural organizations and mechanical interactions between a limited number of molecular domains. This includes force-triggered conformational changes, as well as binding and unbinding events between receptors and ligands [[27], [28], [29], [30]]. However, due to computational constraints, MD models are usually confined to short timescales, typically within several microseconds. On the other hand, MC models divide membranes and substrates into ordered lattices, representing mechanosensitive receptors as simplified pieces positioned on the lattice nodes [31,32]. This approach enables a coarse-grained depiction of the collective behaviors of multiple receptors on larger spatial and temporal scales, such as clustering. In this section, we provide a brief overview of simulating both cell-extracellular matrix (ECM) and cell–cell interactions using MD and MC models, respectively.

### 2.1 Models for cell–cell interactions

Cell–cell interactions are primarily established through cadherin-mediated adherens junctions, which not only bind cells together but also function as mechanotransducers [[33], [34], [35], [36]]. Adherens junctions can be classified into three types based on their shapes and stability: (i) zonula adherens, (ii) punctum adherens, and (iii) multicellular adherens [37]. The stability of adherens junctions is maintained by calcium-dependent adhesion molecules called cadherins [38]. Cadherin is a superfamily that consists of various members, including classical cadherins, desmosomal cadherins, and protocadherins (Pcdhs) [39]. Classical cadherins have five extracellular repeats (EC1-EC5), a transmembrane domain, and an intracellular domain [40]. Based on their ectodomain sequence, classical cadherins can be further divided into two subfamilies: (i) type I, which includes E-cadherin and N-cadherin, characterized by a HAV peptide motif and a conserved tryptophan residue (Trp2), and (ii) type II, which includes VE-cadherin, characterized by two conserved tryptophan residues (Trp2 and Trp4) but lacking the HAV motif [41]. These classical cadherins form two types of interactions. The first is *trans* interaction, which connects cadherins on two adjacent cells, and the second is *cis* interaction, which connects cadherins on the same cell [33]. *Trans* interactions, facilitated by a tryptophan exchange between two EC1 domains, can adopt two distinct conformations: strand-swap (S-dimer) and X-dimer [42]. The lower-affinity X-dimers preferentially transform into the more stable S-dimers, and this transformation process is dependent on actomyosin-generated tensile forces [43,44]. On the other hand, *cis* interactions aid in cadherin clustering through both extracellular and intracellular domains [33]. Extracellular *cis* interactions (EC-*cis* interactions) occur between the EC1 domain and EC2-3 domains of classical cadherins [45]. However, cadherin clusters formed solely based on EC-*cis* interactions are small and unstable [33]. The instability of EC-*cis* interactions can be significantly reinforced by the formation of an intracellular cadherin-catenin complex (CCC), which relies on *trans* binding and links the clusters to actin filaments [46,47].

**Fig. 2:**
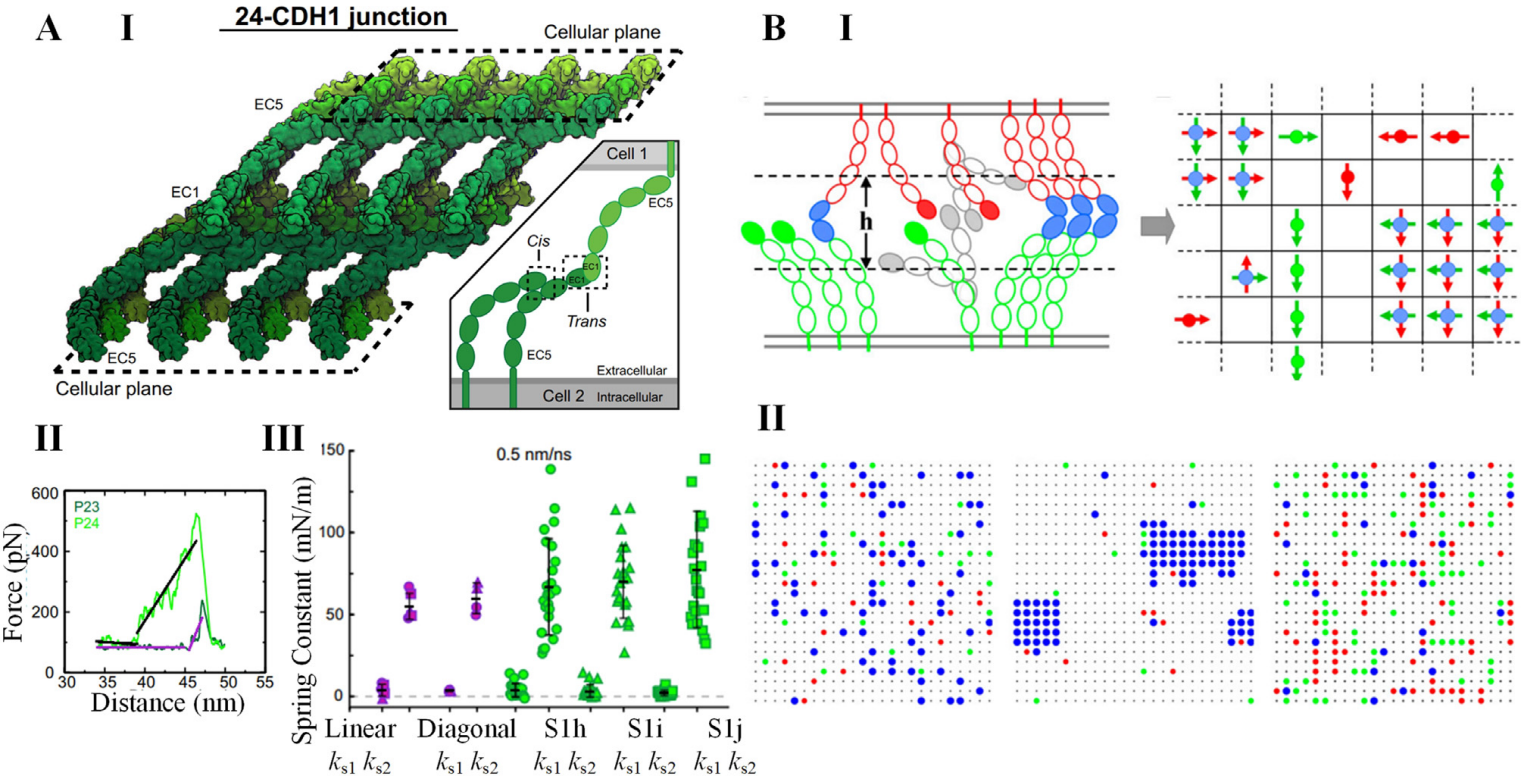
Models for cadherin-mediated cell–cell interactions. **A.** A representative MD model for cadherin. **(I)** The scheme of the MD model depicting extracellular interactions in a 24 ​E-cadherin (CDH1) adherens junction. The insert shows the two types of cadherin interactions. **(II)** The representative force–displacement curves of two opposing cadherin monomers in an individual *tans*-dimer when stretching the two intracellular ends of the 24 ​E-cadherin junction. The light and dark green curves are the simulation results, while purple and black lines are linear fitting. There are two obvious elongation phases in a single force–displacement curve. **(III)** Statistical results of spring constants of cadherin *trans*-dimer, which are calculated from the slopes of the fitting lines as shown in **II**. $k_{s1}$ and $k_{s2}$ denote the spring constants of unbending and unbinding phases respectively. Purples dots are from isolated monomers, while green dots marked S1h-j are from independent replicate simulations of the 24 ​E-cadherin junction. The simulation results indicate *cis* interactions increase the stiffness of adherens junction. **B.** A representative MC model for cadherin. **(I)** The scheme of the MC model depicting cadherin clustering. Left: the lateral view of two cadherin-decorated membranes. Green and red solid ellipses are free EC1 domains of cadherins on the bottom and top membranes respectively. Blue solid ellipses are bound EC1 domains of cadherins. Right: the 2D lattice depicting top view of cell membranes. Green and red dipoles denote cadherins on top and bottom layers respectively. Blue dipoles denote the *trans*-bound cadherin dimers. The *cis* interactions only occurs between dipoles with the same directions. **(II)**. The snapshots show the cadherin patterns formed in different *trans* and *cis* binding affinities. Left: strong *trans* interaction but weak *cis* binding. Medium: both *trans* and *cis* interactions are strong enough. Right: strong *cis* interaction but weak *trans* binding. **A** is adopted from reference [50]. **B** is adopted from reference [61].

MD models have played a crucial role in advancing our understanding of cadherin-mediated cell–cell interactions. These models provide valuable insights into the mechanical responses and molecular structures of cadherin complexes, which are often difficult to detect through traditional experimental methods. By encompassing all the atoms in cadherins, MD models offer atomic-level movies that capture the dynamic relationships between mechanical properties and molecular structures. For instance, these models have revealed that the *trans* interfaces of cadherins exhibit diverse conformations, expanding our understanding beyond the static snapshots provided by experimental crystal structures [48]. By simulating the behavior of individual trans-dimers as well as large complexes, MD models provide valuable insights into their distinct mechanical properties (Fig. 2A) [49,50]. Importantly, the results obtained from MD models can be validated through experimental examinations. For example, MD models have elucidated the molecular mechanisms underlying calcium-dependent catch bond characteristics of X-dimers, which have been confirmed by single-molecule force experiments [51]. Additionally, the combination of MD simulations and single-molecule atomic force microscopy has shed light on how the monoclonal antibody 19A11 strengthens S-dimers through the formation of a salt bridge [52]. Overall, the integration of MD models and single molecular experiments has significantly deepened our understanding of the molecular mechanisms involved in cadherin interactions.

While MD models excel at depicting precise mechanical interactions between isolated cadherin ectodomains, they have limitations when it comes to capturing the entire process of cadherin interactions. This includes factors such as tensile and propulsive forces generated by actomyosin [[53], [54], [55], [56], [57], [58]], cooperativity between *cis* and *trans* bonds [47], cadherin turnover [59], and Rho signaling [60]. This is where MC models come into play, providing a powerful tool for integrating these additional factors and depicting the time-evolutionary patterns of cadherins on a larger spatiotemporal scale [[61], [62], [63], [64]]. These models consider various key processes, including the diffusion of cadherin monomers and dimers, as well as the kinetics of *trans* and *cis* interactions [[61], [62], [63], [64]]. Adherens junction electron micrographs suggest that cadherin assemblies exhibit anisotropic crystal structures [65]. Building upon this understanding, MC models treated specific *cis*-interactions as lateral bindings between two homodirectional dipoles in the same membrane plane, while the two opposing dipoles in a *trans*-dimer are perpendicular to each other (Fig. 2B, I) [61]. The simulation results revealed that both *trans* and *cis* interactions are essential for junction formation (Fig. 2B, II) [61]. Based on this presumption, the multistep kinetic reactions, such as *trans*- and *cis*-dimerization, transition between X- and S- dimer, and alignment of two encountered *trans*-dimers, have been incorporated into the MC models [62]. The kinetic MC model showed that the *cis*-interaction assists formation of *trans*-bond, and glycosylation regulates such a kinetic process [62]. Further studies combining MC models and experiments have suggested that non-specific *cis*-interactions also contribute to cadherin clustering [63]. In addition to the interactions among cadherins themselves, recent MC models have explored the role of the actomyosin cytoskeleton as a crucial regulator of cadherin clustering [64,66,67]. These models indicate that the actomyosin cytoskeleton controls cadherin clustering through spatial confinement and the Rho signaling loop. Thus, MC models provide complementary insights into the complex interplay of multiple molecules within cell–cell junctions, offering us a deeper understanding of cadherin interactions that goes beyond what MD models can provide.

### 2.2 Models for cell-ECM interactions

**Fig. 3:**
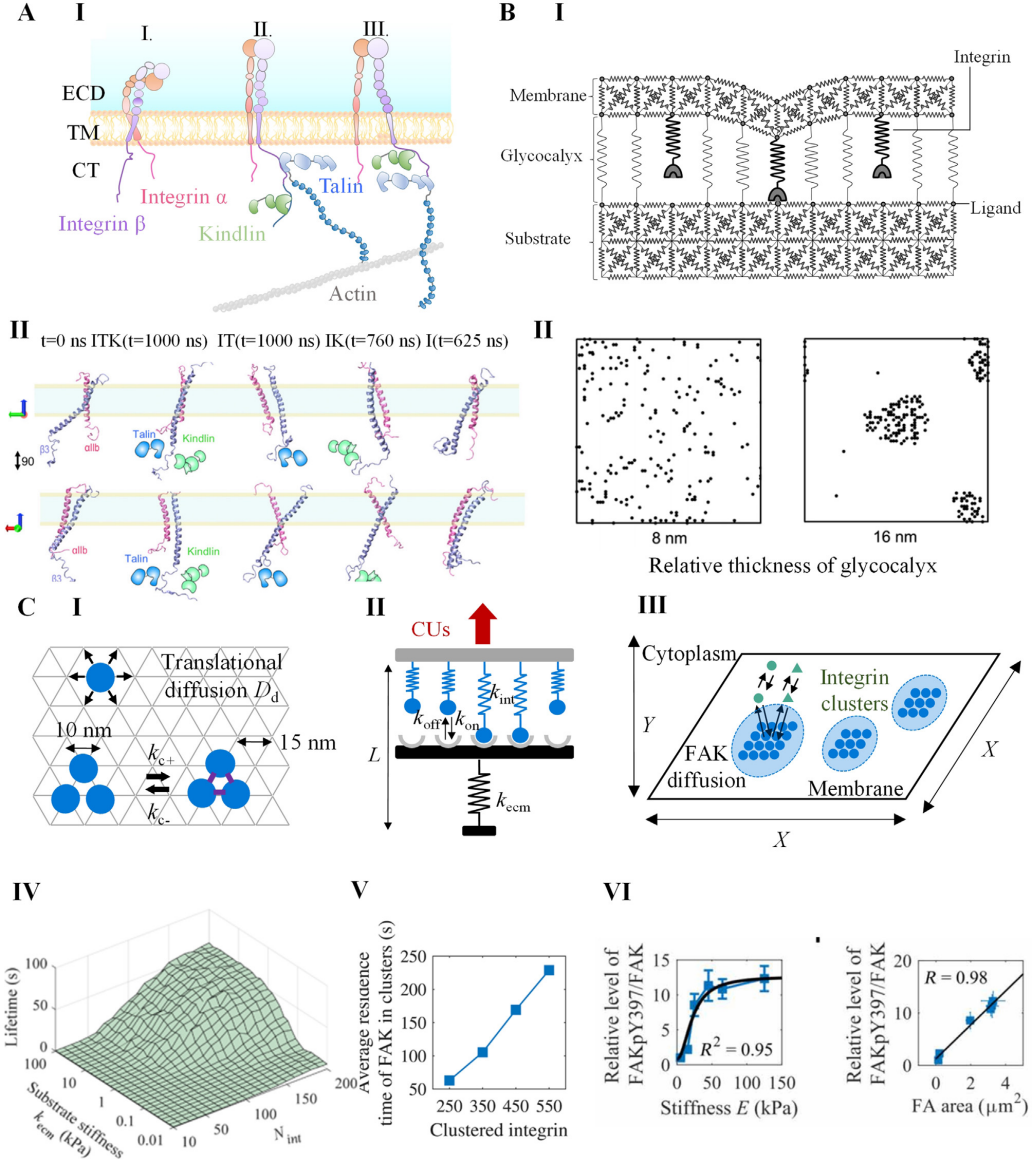
Models for integrin-mediated cell-ECM interactions. **A.** A representative MD model for cadherin. **(I)** Scheme of conformational changes during integrin activation. i-iii denote integrins in bent closed, extended closed, and extended open (activated) states respectively. **(II)** The simulated integrin conformations by the MD models under different scenarios ITK (integrin-talin-kindlin), IT (integrin-talin), IK(integrin-kindlin) and I (integrin alone). The simulation resuts showed the distinct roles of talin and kindling in integrin activation. **B.** A representative MD model for cadherin. **(I)** Scheme of the MC model depicting integrin mediated cell-ECM interactions. In the model, the cell membrane and substrate are set as 3D networks consisted of springs with fixed elastic constants. The cell membrane and substrate are spaced by a glycocalyx layer with a fixed spring constant. Integrins are set as springs diffusing on the cell membrane, which reversibly binding to ligands on the substrate. **(II)** The integrin pattern obtained by the MC model at different relative thickness of glycocalyx. The model showed that increasing glycocalyx thickness assists the integrin clustering by a “kinetic trap” effect. **C.** An integrated model for integrin-initiated mechanotranduction incorporating: the spatial MC model considering kinetics of activation, binding and clustering **(I)**, the spring model considering force-dependent disassembly of integrin **(II)**, and the particle model for spatial arrangement of integrin and FAK molecules **(III)**. The simulation results demonstrated that: the lifetime of integrin clusters is longer on stiffer substrate **(IV)**, and the residence time for FAK in focal adhesion increases with increasing number of clustered integrins **(V)**. These conclusions together explained how substrate stiffness converted to FAK phosphorylation **(VI)**. **A** is adopted from reference [75]. **B** is adopted from reference [83]. **C** is adopted from reference [86].

Cells primarily interact with the ECM through integrin-mediated adhesions [68]. Integrins are composed of a pair of subunits, namely α and β, which are non-covalently connected [69]. Each subunit consists of three domains: a large ectodomain, a transmembrane helix, and a short cytoplasmic tail. There have been identified at least 18 types of α subunits and 8 types of β subunits, which combine to form 24 types of integrin heterodimers [70]. Integrins undergo a conformational change, transitioning from a bent closed and resting state to an extended closed state upon interaction with intracellular molecules, a process known as activation (Fig. 3A, I) [71]. Once activated, integrins bind to ligands on the ECM and recruit other integrins and adaptor proteins to form complex protein clusters connecting actomyosin cytoskeleton [17,72]. The quantitative analysis of these processes can be performed using both MD and MC models, which provide valuable insights into the behavior of integrins.

Like cell–cell junction, MD models also provide valuable atomic-level insights into the dynamics of integrins mediated cell-ECM interactions [27]. These models suggest that integrins maintain a closed state primarily through the inner membrane clasp (IMC) domain on the cytoplasmic tail of integrins, which serves as the trigger of integrin activation [73]. Activators like talins activate facilitate inside-out integrin activation by unclasping the IMC [74]. Kindlin assists in this process by reinforcing the binding between talin and integrin and disrupting salt bridges between integrin α and β subunits (Fig. 3A, II) [75]. Meanwhile, MD models have revealed that certain actin-binding proteins can have dual roles in the talin-mediated integrin activation. For example, α-actinin competes with talin for binding to β3-integrin, thereby impairing integrin activation [76]. However, it also promotes talin binding to β1-integrin by restricting its cytoplasmic tail [76]. Furthermore, MD models have shed light on the role of vinculin, a mechanosensitive adaptor protein, in the integrin-mediated focal adhesion [77]. These models have elucidated how talin binds to and activates vinculin [78,79], as well as how vinculin acts as an actin-binding switch through conformational changes that depend on mechanical load [80]. In addition, MD simulations have also been utilized to investigate how integrins bind to their ligands, such as the RGD peptide [81]. While MD models significantly contribute to our understanding of cell-ECM interactions at the atomic level, they do have limitations. The inclusion of more molecules in simulations incurs substantial computational costs, and the lack of solved molecular crystal structures can restrict the accuracy and scope of MD simulations.

In contrast, MC models simulate the dynamic cell–cell interactions by abstracting away the detailed structural information of adhesion molecules and instead treating them as random-walking entities on membrane lattices [82]. These models simplify the activities of integrins into several kinetic steps: diffusion, activation and deactivation, binding and unbinding to the ECM, and aggregation and separation, providing a more macro level insight. For example, Paszek et al. developed an MC model to study influence of glycocalyx on cell-ECM interactions, where the membrane and ECM were represented by spring-constituted plates (Fig. 3B, I) [83]. They found that thick and stiff glycocalyx facilitated integrin clustering through a “kinetic trap” effect, where the energy barrier for integrin-ECM binding is largely decreased around the bound integrin (Fig. 3B, II) [83]. This finding is consistent with experimental observations that cancer cells expressing more glycocalyx can enhance integrin-mediated growth and survival [84]. Another study by Yu et al. utilized an MC model to investigate the effects of nanoscale ligand spacing and substrate stiffness on integrin clustering [85]. Their modeling results suggested a certain threshold of ligand spacing (typically 60 ​nm) for integrin clustering [85]. In addition, integrin-mediated focal adhesion is an essential converter between the mechanical inputs and biochemical signaling. To understand how integrin clusters undergo the mechanochemical conversion, our group developed an integrated MC model consisting of three parts: an MC model for integrin clustering, a spring model for force-dependent disassembly of integrin clusters, and a particle model for cluster-dependent phosphorylation (Fig. 3C, I-III) [86]. The model showed that the stiffness-enhanced phosphorylation of focal adhesion kinase (FAK) by forming more stable integrin clusters rather than affecting the interactions between focal adhesion kinase (FAK) and integrin clusters (Fig. 3C, IV-VI) [86,87]. These MC models have identified key factors influencing clustering and elucidated the mechanotransduction processes initiated by integrin clusters.

## 3 Models for actin and myosin in cytoskeletons

Cytoskeleton, composed of actomyosin, microtubules, and intermediate filaments, is responsible for load bearing in cells [7,88,89]. Among these components, actomyosin, which consists of actin and myosin, plays a crucial role in responding to mechanical forces and generating active forces that allow cells to adhere, spread, migrate, divide, and sense their environment [90]. Actomyosin generates forces through two main mechanisms: protrusion and contraction [91]. Protrusion is initiated by actin polymerization, which is regulated by various force-sensitive nucleators (*e.g.* formins [92,93], VASP [94,95] and Arp2/3 [96,97]), and terminated by serving and capping proteins (*e.g.* ADF/cofilin [98,99]). On the other hand, contraction is driven by myosin, which is activated by mechanosensitive receptor-triggered signaling cascades involving molecules (*e.g.* calcium [100], Rho [101], ROCK [100]). Through these force generation processes, actomyosins form interconnected and interconvertible structures such as filopodia, lamellipodia, stress fibers, and the cell cortex [7]. These structures collectively create a contractile network that connects the cell membrane to the nucleus. To understand how actomyosin structures adapt to the complex mechanical environment, various mathematical tools have been developed. Over time, these models have evolved to capture the intricate behaviors of actomyosin and its response to mechanical cues. In the following section, we will provide a brief introduction to different actomyosin structures and touch upon the evolution of models used to describe their dynamics.

### 3.1 Filopodia

Filopodia are thin, finger-like membrane protrusions formed by crosslinked bundles of actomyosin [102]. They play a crucial role in various cellular processes such as cell adhesion, migration, and sensing the mechanical properties of the environment [103]. The contraction generated by filopodia involves a “motor-clutch” machinery [104]. In this machinery, myosin motors generate forces that cause the actin filament within the filopodium to retrograde or move backward from the integrin clutches. Simultaneously, the integrin clutches engage and dissociate from the actin filament in a dynamic manner [105]. This motor-clutch machinery enables cells not only to exert mechanical forces on the substrate, but also to probe and sense their mechanical environment. This mechanical probing is essential for processes such as cell migration and the response to external stimuli.

**Fig. 4:**
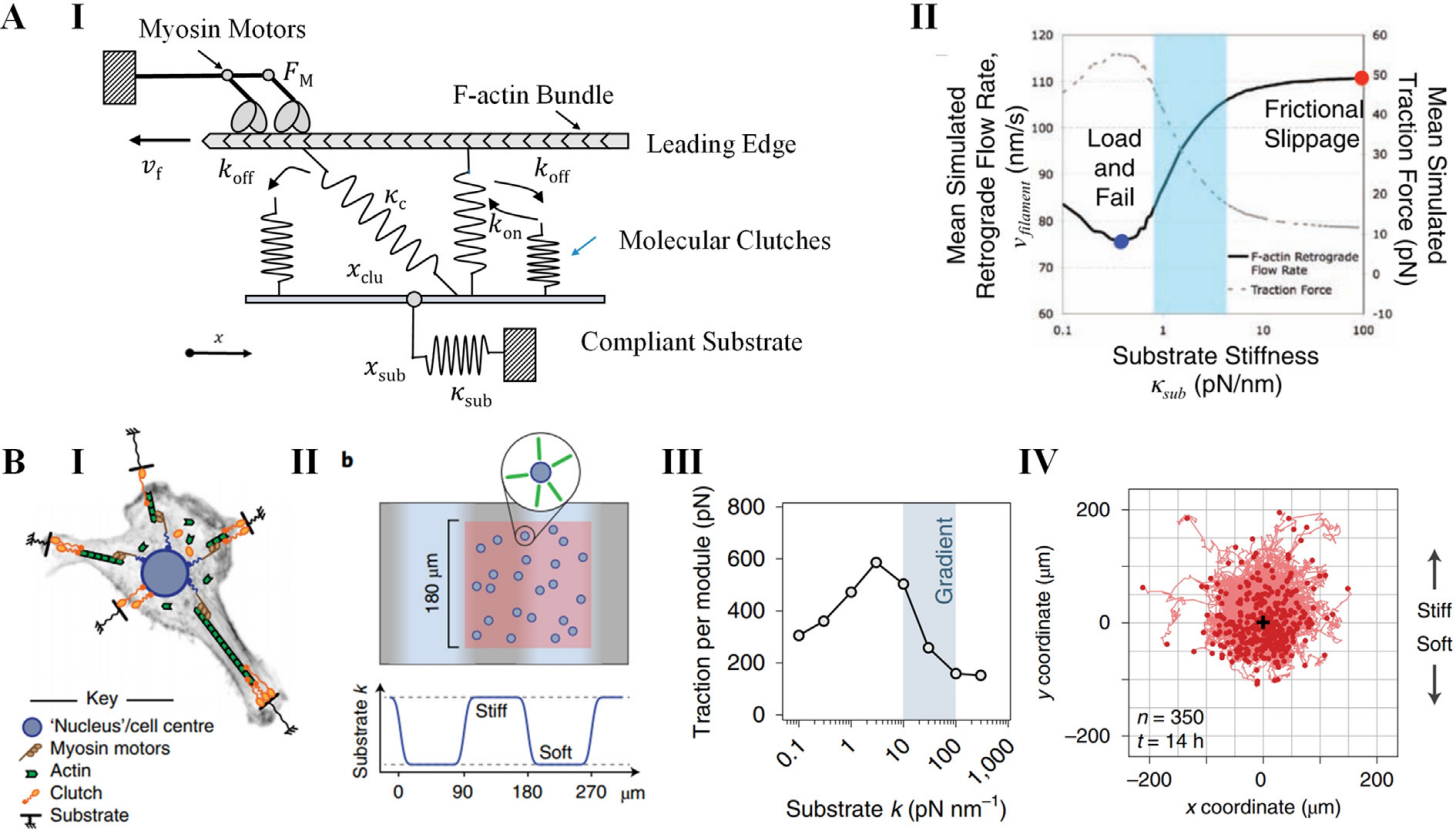
Models for filopodia contraction. **A**. A motor-clutch model depicting cellular contraction generated by filopodia. **(I)** The schematic illustration of the model. The actin filaments bind to the substrate by the molecular clutches. The myosin motors drive the actin filament slipping from the substrate. The substrate and clutches are elongated during loading. **(II)** The simulation results by the model predicted that traction force (dashed gray line) decreases while actin retrograde flow rate (solid black) increase as substrate stiffness increasing. **B**. A cell migration simulator based on motor-clutch model. **(I)** The schematic illustration of the model. In the model, the actomyosin filaments are modeled as motor-clutch modules, and the central cell body is attached to the substrate by a set of clutch molecules. The join force provided by actomyosins drive the cell migration. **(II)** Simulating cell migration on a stiffness-gradient substrate alternating soft and stiff regions. **(III)** The simulated average traction forces of modules as a function of substrate stiffness. The traction force is larger on the soft region of the stiffness-gradient substrate. **(IV)** The simulated tracks of the cells migrating on the stiffness-gradient substrate. **A** is adopted from reference [105], **B** is adopted from reference [114].

To mathematically depict the “motor-clutch” mechanism, Chan and Odde introduced a stochastic physical model. In this model, molecular clutches and the substrate are represented as simple Hookean springs with respective spring constants $\kappa_{\text{clu}}$ and $\kappa_{\text{sub}}$ [105]. The model considers an actin filament with $n_{m}$ motors that drives retrograde flow at a velocity $v_{f}$ (Fig. 4A, I) [105]. Additionally, the integrin clutches engage the actin filament reversibly with rate constants $k_{\text{on}}$ and $k_{\text{off}}$ [105]. The dissociation rate $k_{\text{off}}$ of the engaged clutch is dependent on the force applied to it ($F_{\text{clu}}$) according to a Bell model as [105]:

(1) $$k_{\text{off}}=k_{\text{off}0}\;\exp\left(F_{\text{clu}}/F_{b}\right)$$

where $k_{\text{off}0}$ is the dissociation rate of unloaded clutch, $F_{b}$ is characteristic force of bond rupture. The velocity of retrograde flow $v_{f}$ can be calculated according to a linear force–velocity relation as [105]

(2) $$v_{f}=v_{u}\left(1-\frac{\kappa_{\text{sub}}x_{\text{sub}}}{n_{m}F_{m}}\right)$$

where $v_{u}$ is the sliding velocity of unloaded actomyosin, $F_{m}$ is the stalling force of a single myosin motor. The displacement of substrate can be updated according to the force balance between the clutches and the substrate is given by [105]

(3) $$x_{\text{sub}}=\frac{\kappa_{\text{clu}}\sum_{i=1}^{n_{\text{eng}}}x_{i}}{\kappa_{\text{sub}}+n_{\text{eng}}\kappa_{\text{clu}}}$$

where $n_{\text{eng}}$ is the number of the engaged clutches.

The motor-clutch model proposed by Chan and Odde has been successful in depicting the stiffness-dependent behaviors observed in growth-cone filopodia. It explained why softer substrates, larger traction forces and slower retrograde flow are observed (Fig. 4A, II) [105]. However, it is important to note that different cells may exhibit diverse stiffness–responsive profiles. To explain these variations, the motor-clutch model has been modified in several ways. These modifications consider factors such as the number of clutches and motors [106], different types of integrins [107], talin-reinforced integrin clutches [108], and the kinetics of Rho signaling [109]. Furthermore, the motor-clutch model has been extended beyond the behavior of a single actin filament bundle. It has been used to explain other phenomena, such as ligand distribution-dependent adhesion growth [110], the antagonism between cadherin and integrin [111], and cell migration on substrates with different mechanical properties (*e.g.* uniform elasticity [112], and viscoelasticity [113]). Based on the motor-clutch model, our group also proposed a cell migration simulator considering the cell as an assembly of multiple motor-clutch modules (Fig. 4B, I). We investigated the cell migration on substrate with stiffness gradient (Fig. 4B, II) [114]. The simulation results indicate an unusual negative durotaxis behavior in cells, where traction force reaches its maximum at the soft region (Fig. 4B, III and IV) [114]. These extended models have provided insights into how cells adapt to their mechanical environments. They highlight the coordinated action of actomyosin bundles and integrin-mediated adhesions in governing cellular responses and behaviors.

### 3.2 Lamellipodia

Lamellipodia are membrane protrusions found at the leading edge of motile cells, which consists of a dendritic network of actomyosin [115,116]. The actin filaments within lamellipodia undergo three distinct steps in their life cycle. First, they are “born” through dendritic nucleation, where new filaments emerge from existing ones. Next, these filaments elongate through actin polymerization, extending the lamellipodium further. Finally, the elongation of filaments is terminated by capping proteins, which prevent further growth [117]. During nucleation, the branch angle between the daughter filament and the mother filament is maintained at approximately 70° [117]. This angle is determined by the nucleator called the Arp2/3 complex, which plays a crucial role in lamellipodia formation [118]. Importantly, the nucleator Arp2/3 complex is highly sensitive to forces [118]. This characteristic allows lamellipodia to adapt to and exert pushing forces on the surrounding mechanical environment.

**Fig. 5:**
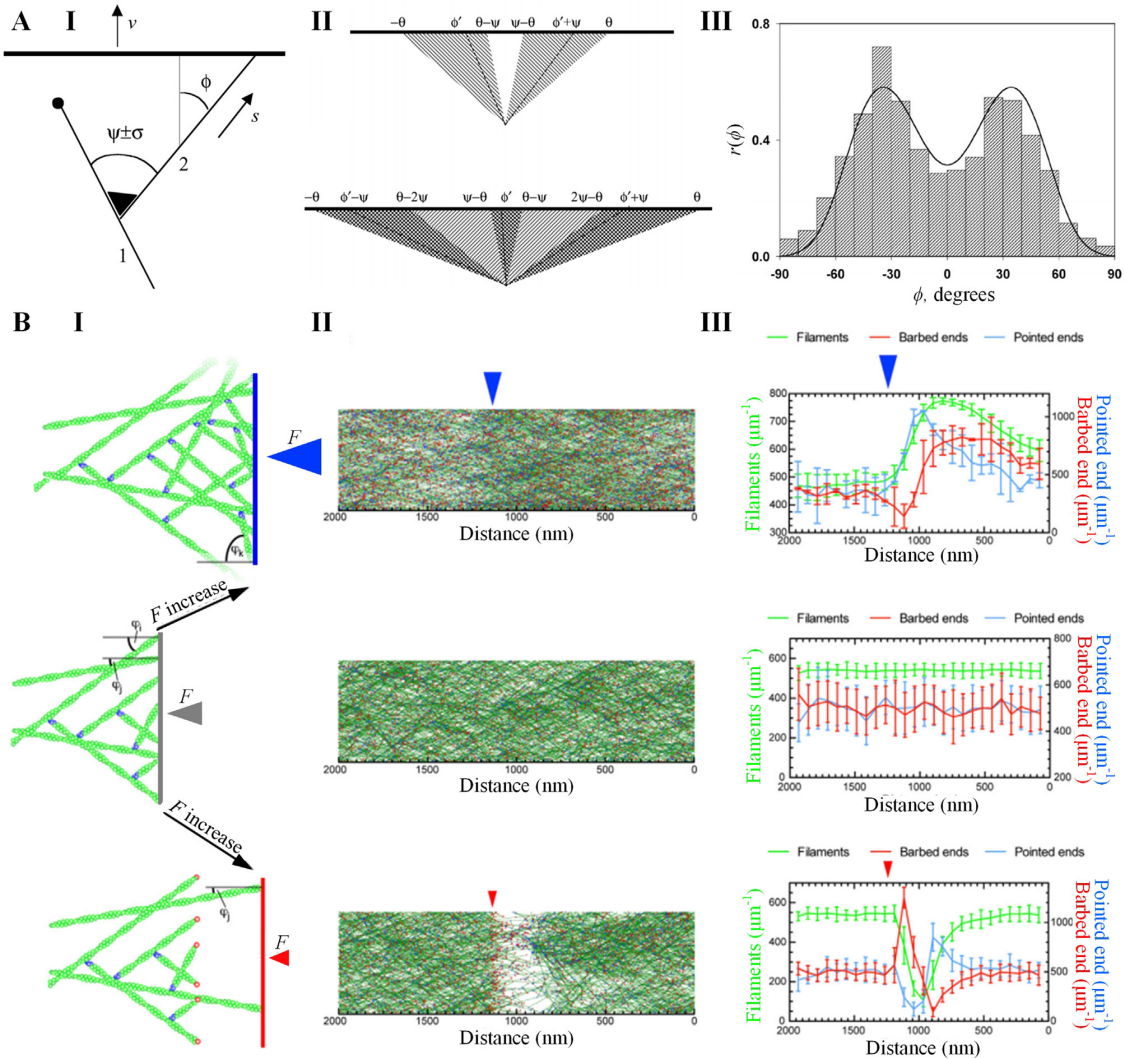
Models for lamellipodia protrusion. A. A population–kinetics model for propelling leading edge of motile cell by dendritic nucleation. **(I)** The schematic illustration of the model. Near the leading edge, the new filament are nucleated on the existing filament and push the membrane advance. **(II)** Two reproductive patterns of actin filaments predicted by the model: when ψ/2<θ≤ψ, filaments reproduce in two orientations (top), when θ>ψ, generating filaments in three orientations become possible (bottom). (**III**) The comparison between theoretical prediction (line) and electron micrograph statistics (histogram) of filament orientations at the leading edge. **B**. A stochastic model simulating the lamellipodia protrusion against applied loads. **(I)** The schematic illustration showing filament growth at a steady state against an intermediate load (middle, gray arrow), after increasing the load (top, blue arrow) and decreasing the load (bottom, red arrow). The barbed end and pointed end of filament are marked as red and blue respectively. Changing the load affects the filament density and angle distribution. **(II)** Simulated filament distributions under the three load conditions. **(III)** Quantification of filament numbers, barbed, and pointed end density under the three load condition. The simulation results suggested that increasing load leads a nucleation peak and an increase in filament density, while decreasing load increases capping, and decreases nucleation and thus filament density. **A** is adopted from reference [117]. **B** is adopted from reference [91].

To quantitatively analyze the actin branching in lamellipodia protrusion, Maly and Borisy introduced a population-kinetic model (Fig. 5A, I) [117]. In this model, the angle between a newly nucleated filament and its mother filament follows a distribution with a mean angle ψ and a standard deviation σ. The protrusion of the membrane occurs as filaments grow and push against it at a velocity v. The orientation of each filament is characterized by its incidence angle φ with respect to the membrane (Fig. 5A, II) [117]. Filament elongation is achieved by the addition of actin monomers at a rate s, which can be calculated by [117]

(4) $$s=\delta k_{a}M_{a}p$$

where δ is the distance contributed by adding a single actin monomer, $k_{a}$ is the rate constant of actin polymerization, $M_{a}$ is the concentration of actin monomers, and p is the probability that the filament is open rather than obstructed by the membrane. For a filament at edge, v=s·cosφ, and $p=p_{0}/\cos\;\varphi$, where $p_{0}=v/\left(\delta k_{a}M_{a}\right)$ is the probability that the filament perpendicular to membrane is open. When |φ| increases larger than a critical angle $\theta =\arccos\;p_{0}$, the filament is excluded from the model since it loses contact with the protruding membrane. For filaments that have an open configuration with |φ|<θ, the actin polymerization can be terminated at a capping rate $cp=cp_{0}/\cos\;\varphi$, where c is the capping rate constant. Then, the time-evolving orientation distribution at the leading edge of cell can be deviated as [117]

(5) $$\frac{dn\left(\varphi ,t\right)}{dt}=\frac{b}{\sqrt{8\pi }\sigma }\int_{-\theta }^{\theta }\left(\exp\left(-\frac{{\left(\omega +\psi -\varphi \right)}^{2}}{2\sigma^{2}}\right)+\exp\left(-\frac{{\left(\omega +\psi -\varphi \right)}^{2}}{2\sigma^{2}}\right)\right)n\left(\omega ,t\right)d\omega -\frac{cp_{0}}{\cos\;\varphi }n\left(\varphi ,t\right)$$

where n(φ,t) is the number of filaments at orientation φ and time t, b is the born rate of actin filaments.

Agreeing with experimental statistics, the simulation results of the Maly-Borisy model suggest that the orientation of actin filaments at leading edge is naturally selected into ±35° or +70°/0°/−70° patterns at the steady state (Fig. 5A, III) [117]. However, since the stochastic nature of the 2D model by Maly and Borisy simplifies many essential mechanical details in the development of lamellipodia, such as membrane and filament mechanics, membrane tension, and mechanical load by barriers, continuous efforts have been devoted to improve the Maly-Borisy model to investigate lamellipodia in more complex conditions. By incorporating diffusion, stochastic kinetics, and elasticity of the membrane and filaments into the Maly-Borisy model, Schaus et al. demonstrated that the orientation pattern of actin filaments is highly sensitive to the values of the context parameters, such as the velocities of membrane protrusion and actin polymerization, as well as filament bending lengths [119]. Furthermore, considering that Arp2/3 can be activated by bending filaments, Weichsel and Schwarz showed that the transition between ±35° and +70°/0°/−70° patterns leads to a hysteresis effect in the force-dependent velocity of protrusion, which is responsible for the convex and concave shapes of the membrane [120]. Based on these earlier work, Mueller et al. developed a stochastic model of lamellipodia growth against different external forces, and revealed that the protruding network of lamellipodia adapts to load by adjusting geometry of branched actin and protrusive force (Fig. 5B) [91].

### 3.3 Stress fiber

Stress fibers are aligned actomyosin bundles distributed in the cytoplasm that interact with focal adhesions to generate the main contractile forces on ECM [[121], [122], [123]]. They not only play an important role in mechanotransduction, but also have the ability to adapt to various mechanical stimuli (*e.g.* cyclic loading [124], patterned adhesions [125], and dynamic adhesions [126]) by rearranging their orientations. Understanding how stress fibers generate and reorient under these mechanical stimuli has become a prominent research topic.

**Fig. 6:**
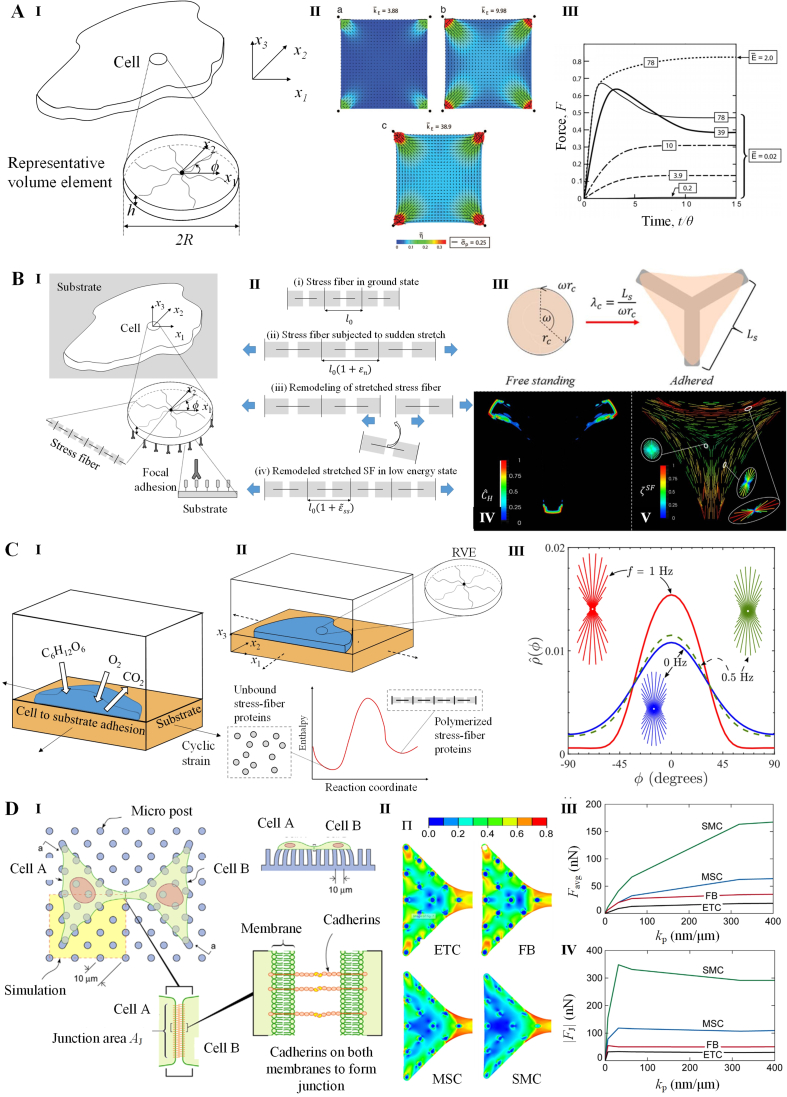
Models for stress fiber dynamics. **A**. A bio-chemo-mechanical model depicting assembly and contraction of stress fibers. **(I)** The model scheme illustrating a 2D cell consisting of a network of stress fibers. **(II)** The simulated distribution of stress fibers activation level (η) in cells on the micro-posts with varied stiffness ($k_{E}$). **(III)** The time evolution of normalized traction force provided by the cells on micro-posts with varied stiffness. The simulation results demonstrate that the activation level of stress fiber and thus cellular traction force increase with the support stiffness. **B**. An extended model depicting stress fiber behaviors in cells on ligand-patterned substrates. **(I)** The model scheme illustrating in a 2D cell on ligand-coated substrate. Both stress fibers and focal adhesions are considered in the representative volume element (RVE) of the cell. **(II)** The model scheme illustrating remodeling of a stress fiber subject to tensile strain ($\varepsilon_{n}$). **(III)** The model scheme illustrating stretches of the cell on a Y-shaped ligand pattern (gray area). The partial of the cell perimeter ($\omega r_{c}$) in the free standing state is stretched to $L_{S}$ in the adhered state. **(IV)** The simulated distribution of focal adhesions in the spread cell on Y-shaped ligand pattern in the lowest free energy configuration. **(V)** The simulated distribution of stress fiber alignment in the spread cell on Y-shaped ligand pattern in the lowest free energy configuration. These results demonstrate that the cell is spreading toward the state of the minimal free energy through adjusting both the contractility of its active stress fiber and the deformation of its passive components. **C**. An extended model depicting stress fiber behaviors in cells under cyclic stretching. **(I)** The model scheme illustrating a cell adhering to a substrate subjected to cyclic stretch. The cell exchanges substances with the nutrient bath. **(II)** The model scheme illustrating the 2D cell with stress fiber components in unbound and polymerized state. **(III)** The simulated orientation distribution of stress fibers in cells subjected to cyclic stretching with varied loading frequency. The simulation results indicate that increasing cyclic loading results reorientations of cell and its stress fibers. **D**. An extended model depicting cell pairs on micro-post arrays. **(I)** The model scheme illustrating two cells adhering to micro-post connect to each other by cadherin mediated cell–cell junction. **(II)** The predicted stress fiber distribution in the different kinds of cell pairs. ETC: endothelial cell. FB: fibroblast. MSC: mesenchymal stem cell. SMC: smooth muscle cell. **(III-IV)** The predicted average traction ($F_{\text{avg}}$) and junction tugging force ($\left|F_{J}\right|$) of different kinds of cell pairs as functions of micro-post stiffness ($k_{p}$). **A** is adopted from reference [127]. **B** is adopted from reference [132]. **C** is adopted from reference [135]. **D** is adopted from reference [136].

To investigate the assembly and contraction of stress fibers, Deshpande et al. developed a bio-chemo-mechanical model (Fig. 6A) [[127], [128], [129]]. In the model, the formation of stress fiber is initiated by activation signaling, which decays exponentially over time as C=exp(−t/θ), where θ represents the signal decay constant. The maturation of stress fibers is characterized by their activation level η. At a given angle φ, η evolves over time as [127]

(6) $$\frac{d\eta \left(\varphi \right)}{dt}=\left(1-\eta \left(\varphi \right)\right)\frac{C{\bar{k}}_{f}}{\theta }-\left(1-\frac{\sigma \left(\varphi \right)}{\sigma_{0}\left(\varphi \right)}\right)\eta \left(\varphi \right)\frac{{\bar{k}}_{b}}{\theta }$$

where ${\bar{k}}_{f}$ and ${\bar{k}}_{b}$ are dimensionless parameters of stress fiber formation and dissociation respectively, σ is the tension in the stress fiber, and $\sigma_{0}$ is the corresponding isometric stress. And $\sigma_{0}=\eta \sigma_{\max}$, where $\sigma_{\max}$ is the tensile stress exerted by a full activated stress fiber. The stress in stress fiber σ is related to its deformation rate $\dot{\varepsilon }$ through the cross-bridge dynamics depicting by Hill-type function [127]

(7) $$\frac{\sigma }{\sigma_{0}}=\left\{ \begin{array}{c} 0\;\frac{\dot{\varepsilon }}{{\dot{\varepsilon }}_{0}}<-\frac{\eta }{{\bar{k}}_{v}}\\ 1+\frac{{\bar{k}}_{v}}{\eta }\left(\frac{\dot{\varepsilon }}{{\dot{\varepsilon }}_{0}}\right)\;-\frac{\eta }{{\bar{k}}_{v}}\leq \frac{\dot{\varepsilon }}{{\dot{\varepsilon }}_{0}}\leq 0\\ 1\;\frac{\dot{\varepsilon }}{{\dot{\varepsilon }}_{0}}>0 \end{array}\right.$$

where ${\bar{k}}_{v}$ is the fractional reducing rate of stress at fiber shortening rate ${\dot{\varepsilon }}_{0}$.

The Deshpande's model has been successful in capturing key experimental features of stress fibers, including: (i) the increase in contraction forces with higher substrate stiffness, (ii) the influence of cell shape on structural anisotropy, (iii) the concentration of stress fibers around focal adhesions. Furthermore, the model has been extended to study additional aspects of stress fibers in various cellular contexts. These include investigating adhesion, remodeling, and the contractile response of stress fibers in cells cultured on ligand-patterned substrates (Fig. 6B) [[130], [131], [132]], subjected to cyclic stretching (Fig. 6C) [[133], [134], [135]], or adhering to other cells (Fig. 6D) [136]. Besides Deshpande's models, Kaunas et al. have also established kinematic models that support cells remodeling stress fibers to reestablish their tensional homeostasis in response of mechanical stimuli [137,138]. These extensions have helped provide insights into how stress fibers adapt and respond to different mechanical stimuli in diverse biological environments.

### 3.4 Cortex

The actin cortex refers to a thin layer of crosslinked actomyosins that are attached to the cell membrane [[139], [140], [141]]. It plays a crucial role in determining cell shape, polarization, and division. The thickness of the cortex can vary from a few hundred nanometers to micrometers, depending on factors such as cell type, spreading conditions, stage of the cell cycle, and measurement techniques [141]. The mesh size of the cortex is typically around 100–200 ​nm [141]. Despite its relatively low density of actin filaments and an isotropic meshwork, similar to other actin structures, the cortex actively drives protrusive and contractile machineries [141]. However, what sets the cortex apart from other actin structures is its behavior as a viscoelastic gel, capable of undergoing remodeling through cortical flow [142].

**Fig. 7:**
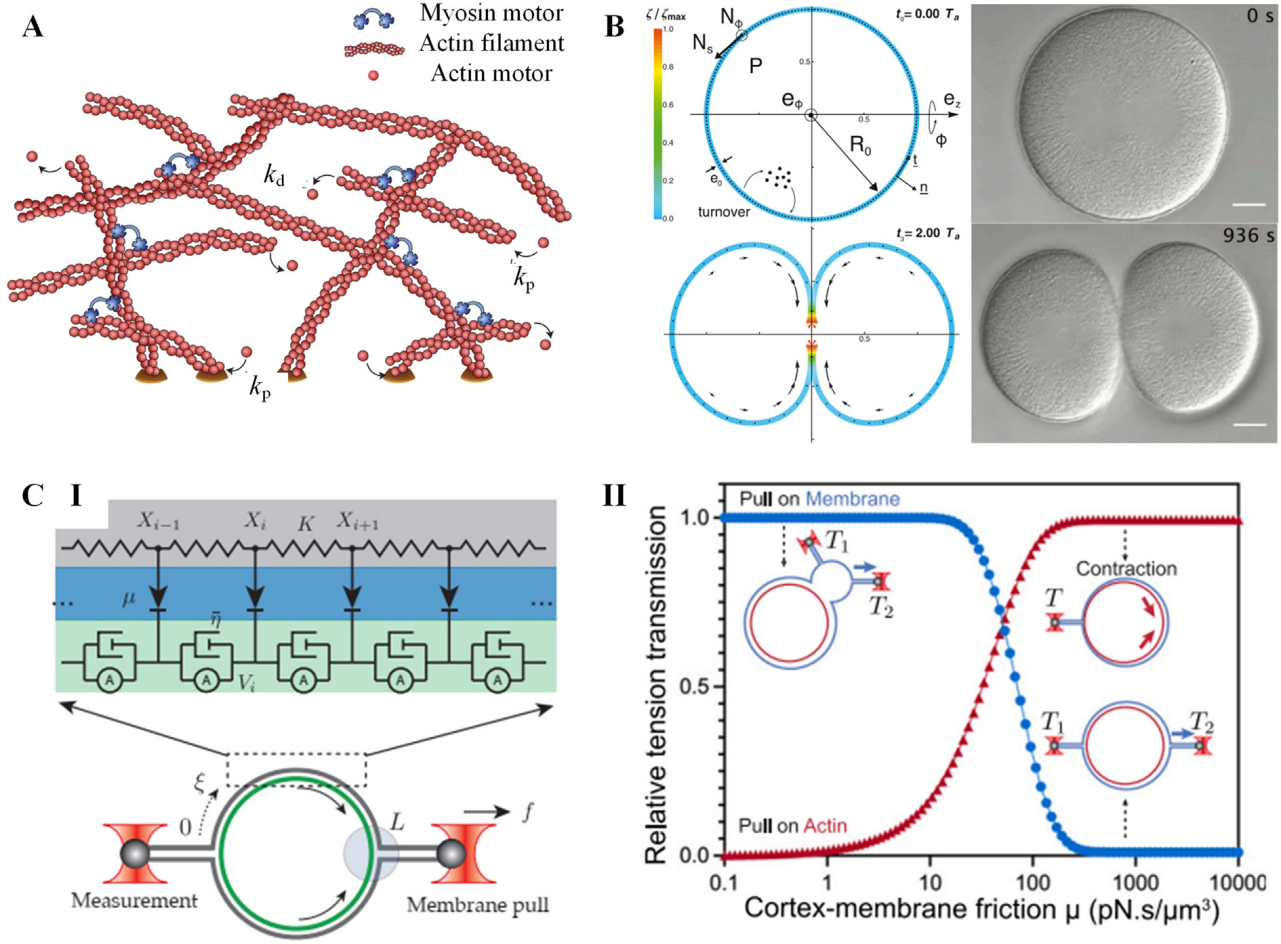
Models for cortex mechanics. **A.** The model scheme illustrating an active gel network consisted of myosin motors, actin filaments and crosslinkers. The actin filaments are continuously polymerizing and depolymerizing at rate $k_{p}$ and $k_{d}$ respectively. **B**. A model based on active gel theory for depicting cortex dynamics during cytokinesis. Left is the simulated evolutions of cell shape and cortex thickness over time. Right is the microscopy images of a dividing cell. The color bar ($\zeta /\zeta_{\max}$) indicates the myosin activity signal. **C**. A 3-tier composite model depicting the membrane tension propagation. **(I)**. The model scheme illustrating mechanical representations of membrane-cortex structure. The membrane is modeled as a series of linear springs (gray area). The adhesive linker between membrane and cortex is modeled as frictional components (blue area). The cortex is modeled as a thin layer of active gel (green area). **(II)** The simulated tension transmission as a function of cortex-membrane fiction for different targets of force application. The results summarizing three modes of membrane tension propagation observed in the experiments. **A** is adopted from reference [143]. **B** is adopted from reference [147]. **C** is adopted from reference [153].

The cortex's network structure can be effectively described using active gel models based on continuum theories [16,143]. In the typical framework of active gel theory, the actomyosin network is represented as a contractile viscoelastic gel immersed in a fluid medium (Fig. 7A, I) [16,143]. The total stress tensor ($\sigma_{t}$) in the network is divided into active part and passive part as [143]

(8) $$\sigma_{t}=\sigma_{a}+\sigma_{p}$$

where $\sigma_{a}$ and $\sigma_{p}$ are active and passive stresses in the actomyosin network respectively. The active part ($\sigma_{a}$) is expressed as [143]

(9) $$\sigma_{a}=\zeta \Delta \mu$$

where ζ is the coefficient dependent on the motor density, and its positive (negative) value correspond to the contraction (expansion) of the system, Δμ is the chemical potential difference resulted from ATP hydrolysis. And the relationship between passive part ($\sigma_{p}$) and the strain is governed by the Maxwell model as [143,144]

(10) $$\left(1+\tau \frac{D}{Dt}\right)\sigma_{p}=2\eta u$$

where D/Dt denotes the corotational derivative of a tensor, τ is the viscoelastic relaxation time of the actomyosin network, η is the viscosity of the actomyosin network, u is the strain rate tensor. The hydrodynamics of monomer flows are governed by the convective and mass conservation law as [143,144]

(11) $$\frac{\partial \rho_{f}}{\partial t}+\nabla J_{f}=k_{p}\rho_{m}-k_{d}\rho_{f}$$

(12) $$\frac{\partial \rho_{m}}{\partial t}+\nabla J_{m}=-k_{p}\rho_{m}+k_{d}\rho_{f}$$

where $\rho_{f}$ and $\rho_{m}$ are the densities of monomers in the filament and solution respectively, $J_{f}$ and $J_{m}$ are the currents of monomer flow in the filament and solution respectively, $k_{p}$ and $k_{d}$ are polymerization and depolymerization rates of actin filaments respectively.

The frameworks of active gel theories have proven successful in extending our understanding of the shape, flow, and stress behaviors of actin cortexes during various cellular processes. These include cytokinesis [[145], [146], [147]], shape oscillations [148], and cell motility [149] (See Fig. 7A, II as an example). As new experimental phenomena emerge, active gel theories for the cell cortex continue to develop by incorporating more complex processes. For example, Yin et al. combined thin shell theories with active gel theories and to explore how biochemical and mechanical signals collaborate in regulating morphogenetic patterns in the cortex [150]. This model successfully captures surface contraction waves and RhoA concentration waves in cells with varying levels of actomyosin expression [150]. Rocha et al. take in account of viscoelastic properties and active turnover of cortex in the active gel theory, which accurately predicts the three-dimensional shape changes of cells during biological events like osmotic shocks and division [151].

Recent studies on the cortex pay close attention to the propagation of membrane tension, which plays a crucial role in cell signaling and mechanosensation. Shi et al. developed a measurement method for membrane tension under external forces and a hydrodynamic model of membrane flow to examine the speed and extent of local tension propagation on the membrane [152]. This model suggested that membrane tension rapidly propagates over long distances on a cell-attached bleb, but it is largely impeded on intact cells due to the presence of cortex-bound transmembrane proteins that restrict its propagation [152]. Building upon this research, Henry et al. discovered that tension generated directly in the cortex through optogenetic control of Rho signaling can also propagate rapidly over long distances [153]. They proposed a mechanical model, the membrane-adhesive linker-cortex 3-tier model, to explain these different tension propagation phenomena observed on cell surfaces (Fig. 7B, I) [153]. According to their model, the long-range membrane response depends on the mechanical forces acting on the membrane and the attachment between the membrane and the cortex, rather than whether the force is endogenously or exogenously applied (Fig. 7B, II) [153]. These mechanical models provide valuable insights and serve as a reference for better understanding the diverse functions of cell cortex, like cortex flow, membrane morphology, and tension propagation.

## 4 Models for dynamics and mechanotransduction of nucleus

Nucleus is known as the “container” of genome and plays a critical role in controlling cellular activities [13,154]. Not only does the nucleus adapt its mechanical properties to the cellular microenvironment, but it also serves as a secondary mechanosensory surface, complementing the plasma membrane [154]. Research has shown that the nucleus responds to various mechanical signals (*e.g.* stretching and compressing by actomyosin [10,155], ultrasound [156,157], and shock waves [[158], [159], [160]]) through deformation and downstream mechanotransduction processes. Mechanical models have been developed to depict how the nucleus deforms according to different load stresses and its structural components, as well as how these nuclear deformations generate biochemical signaling through nuclear mechanotransduction [13]. These models provide powerful tools to understand the mechanical properties of the nucleus and the complex processes of nuclear mechanotransduction triggered by nuclear deformation. By incorporating physical principles, these mechanical models help researchers explore the relationships between mechanical forces, nuclear deformations, and the resulting biochemical responses. They contribute to our understanding of how mechanical cues are sensed and converted into biochemical signals within the nucleus, shedding light on the intricate interplay between mechanics and cellular function. In this section, we will introduce the models developed for mechanical properties of the nucleus and the processes of nuclear mechanotransduction triggered by the nuclear deformation.

### 4.1 Models for mechanical properties of the nucleus

Nucleus is regarded as a viscoelastic and incompressible sphere that consisting of nuclear membrane, lamina, chromatin, and nucleoplasm [161]. The mechanical properties of nuclei vary not only according to the tissue context but also in (patho-)physiological processes. These properties are mainly dependent on the compositions of lamina and the structures of chromatin. The nuclear lamina is an elastic network located beneath the inner nuclear membrane which composed of lamin A, B, and C [161,162]. Lamin A and C are encoded by the same gene (*LMNA*), while lamin B family members are encoded by two genes (*LMNB1* and *LMNB2*) [163]. Therefore, lamin A and C are often referred together as lamin A/C.

**Fig. 8:**
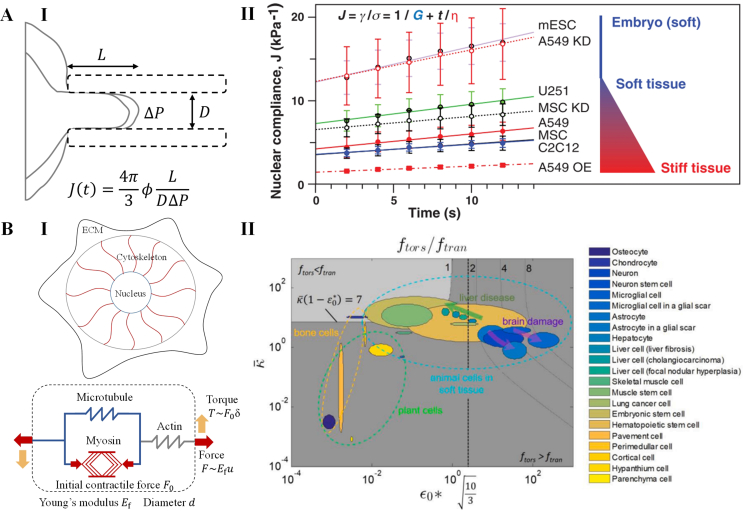
Models for mechanical properties of nucleus. **A**. A model for nuclear compliance measurement. **(I)** The model scheme illustrating how nuclear compliance is measured by aspiration and imaging. **(II)** Nuclear compliances of different cells as a function of time. **B**. A model for vibrational response of nucleus. **(I)** The model scheme illustrating the structure of a cell residing in ECM (Up). The cytoskeleton is modeled as a composite of contractile myosin in parallel with elastic microtubule and in series with elastic actin (Down). **(II)** The phase diagram of cytoskeletal stiffness ($\bar{k}$) and initial strain generated by cytoskeletal filaments ($\varepsilon_{0}^{*}$). The diagram is divided into torsional vibration-dominated regime ($f_{\text{tors}}>f_{\text{tors}}$, dark area) and translational vibration-dominated regime ($f_{\text{tors}}<f_{\text{tors}}$, light area). The natural frequencies of torsional and translational of the nucleus are also marked for a range of cell types. **A** is adopted from reference [161]. **B** is adopted from reference [166].

To quantify the how nuclear components determine mechanical properties of nucleus, Pajerowski et al. developed a measurement method based on microaspiration and fluorescence imaging (Fig. 8A, I) [164]. By applying a constant pressure (ΔP), the nucleus extends into the micropipette (diameter D) over time. The compliance of the nucleus (J) is then determined by the extension length of the nucleus (L) as

(13) $$J\left(t\right)=\frac{4\pi }{3}\varphi \frac{L\left(t\right)}{D}\frac{1}{\Delta P}$$

where t is the relaxation time, Φ=2.1 is a constant. They found that the creep compliance of nucleus falls in a power law as

(14) $$J\left(t\right)=A{\left(t/t_{0}\right)}^{\alpha }$$

where A is a constant with unit of reverse stiffness (Pa^−1^), $t_{0}\equiv 1\;s$, and α∈[0,1] is an index capturing mechanical response from solid (α=0) to fluid (α=1). The nucleus was found to undergo a transition towards a more solid-like behavior after being subjected to loading for 10 ​s, as evidenced by a decrease in the parameter α from 0.4 to 0.6 to 0.2 [164]. Knocking down lamin A/C has been observed to significantly soften the nucleus of differentiated cells, bringing its stiffness closer to that of stem cells. This softening effect is demonstrated by an approximately 2.2-fold increase in the nuclear compliance (J(t)) [164]. On the other hand, the condensation of chromatin through the presence of divalent cations such as Ca^2+^ and Mg^2+^ leads to a substantial increase in nuclear stiffness [164].

Discher et al. used the same measurement method to investigate how nuclear compliance varies in cells from different tissues [161]. They proposed that the time-dependent behavior of nuclear compliance can be depicted by a simpler Maxwell model as [161].

(15) $$J\left(t\right)=\frac{1}{G}+\frac{t}{\eta }$$

where G and η are the elasticity and viscosity of nucleus respectively. The viscous response time of nucleus can be expressed as [161]

(16) τ=G/η

Their findings revealed that the expression level of lamin A correlates with the stiffness of the tissue (Fig. 8A, II) [161]. Additionally, they established scaling laws between the viscous response time (τ), elasticity (G), and the lamin A:B stoichiometry [161]:

(17) $$\tau \propto \;{\left[\text{laminA}:B\right]}^{2.5}$$

(18) $$G\propto \;{\left[\text{laminA}:B\right]}^{0.5}$$

The scaling relationships are consistent with the predictions of the simple polymer theory, suggesting that lamin A functions as a diffusing filamentous protein contributing to nuclear mechanics [165]. These results indicate that nuclei can adjust their mechanical properties by regulating the expression of lamina and the condensation of chromatin in response to the mechanical microenvironment.

The nucleus, being the stiffest and densest organelle in the cell, can also respond to mechanical stimuli through vibrations, as proposed by Liu et al. [166]. They developed a theoretical model to describe the nuclear response to vibrational signals, taking into account cytoskeletal contractility and anisotropy (Fig. 8B, I) [166]. In the model, the nucleus is simplified as an ellipsoidal inclusion within an infinite, isotropic, linear solid. The natural frequencies for torsional ($f_{\text{tors}}$) and translational ($f_{\text{tran}}$) vibrations of nucleus can be deviated from Walpole solutions as following equations respectively [166]

(19) $$f_{\text{tors}}=\frac{1}{2\pi }\sqrt{\frac{K_{\text{tors}}}{I}}=\sqrt{\frac{5}{\pi }\frac{Gr_{n}}{m}}$$

(20) $$f_{\text{tran}}=\frac{1}{2\pi }\sqrt{\frac{K_{\text{tran}}}{m}}=\sqrt{\frac{3}{2\pi }\frac{Gr_{n}}{m}}$$

where $K_{\text{tors}}$ and $K_{\text{tran}}$ are the torsional and translational stiffness of nucleus, I is inertia moment of nucleus, G is effective shear modulus of cytoplasm and ECM, $r_{n}$ is the nuclear radius, m is the mass of nucleus. When considering the nucleus contracted by radially anisotropic actin filaments (number N, Young's modulus $E_{f}$, length l, cross section area A), the expressions of $f_{\text{tors}}$ and $f_{\text{tran}}$ can be modified as [166]

(21) $$f_{\text{tors}}=\frac{1}{2\pi }\sqrt{\frac{20\pi Gr_{n}+\frac{5N}{3}F_{0}\frac{r_{n}+l}{r_{n}l}}{m}}$$

(22) $$f_{\text{tran}}=\frac{1}{2\pi }\sqrt{\frac{6\pi Gr_{n}+\frac{1}{3}N\frac{{E^{\prime }}_{f}A}{l}}{m}}$$

where $F_{0}$ is the initial contractile force of each actin filament, ${E^{\prime }}_{f}=\omega E_{f}$ is effective Young's modulus of the actin filament with corrected by factor ω. Through formula derivations, the ratio of natural frequency of torsional vibration to that of translational vibration is given as [166]

(23) $$\frac{f_{\text{tors}}}{f_{\text{tran}}}=\sqrt{\frac{10+\bar{\kappa }\varepsilon_{0}^{*}}{3+\bar{\kappa }}}$$

where $\bar{\kappa }$ and $\varepsilon_{0}^{*}$ are dimensionless effective stiffness and initial contractility of the entire cytoskeleton respectively. By applying this model, the authors identified the ranges of natural frequencies of torsional and translational vibrations in various animal and plant cells (Fig. 8B, II). They suggested that natural frequencies of torsional and translational vibrations mainly depend on contractility and stiffness of cytoskeletons respectively. The model provides insights into the vibrational properties of nucleus and offers a theoretical basis for vibration-induced nuclear mechanotransduction.

### 4.2 Models for nuclear mechanotransduction

In various (pathy-)physiological processes, the forces acting on the nucleus primarily originate from the mechanical integration of mechanosensitive receptors, the actomyosin cytoskeleton, and the transmembrane linker of nucleoskeleton to cytoskeleton (LINC) complex [167]. The magnitude of these forces depends on the assembly of the actomyosin cytoskeleton, including structures like perinuclear apical actin cables [168]. These forces regulate transcription and thus cell behaviors through directly stretching chromatin or indirectly triggering nuclear translocation of epigenetic and transcriptional factors [11,169].

**Fig. 9:**
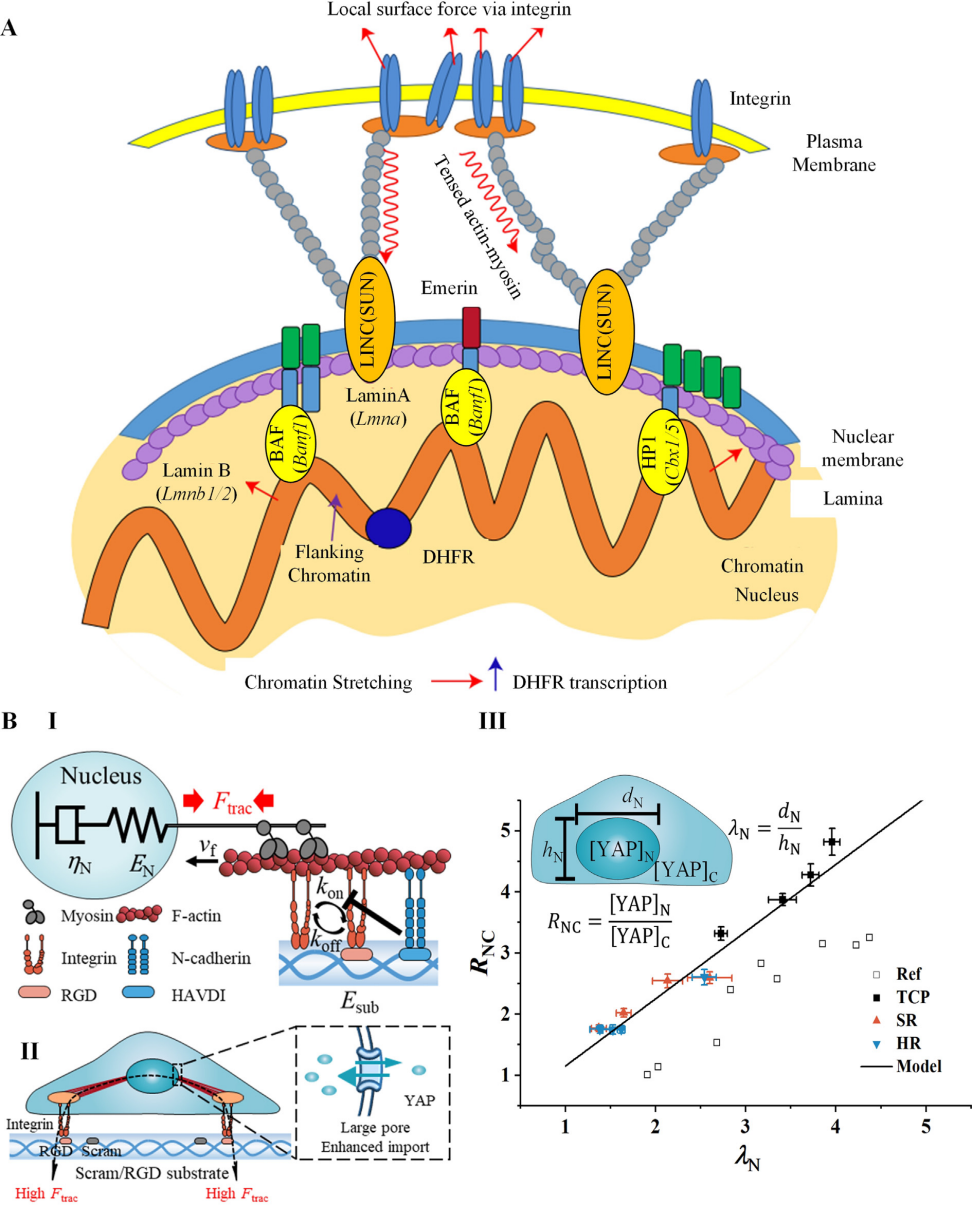
Models for nuclear mechanotransduction. **A.** A model depicting direct force-induced stretching of chromatin. The scheme shows that the external force propagates to nucleus through integrin mediated focal adhesion and cytoskeleton, which stretches the chromatin and upregulate the transcript of DHFR gene. **B.** A model depicting mechanics-driven nuclear localization of transcription factor. **(I)** The model scheme illustrating cadherin disturbs integrin binding and regulate the cellular traction force ($F_{\text{trac}}$) transmitting to the nucleus. The nucleus is regarded as an incompressible viscoelastic sphere. **(II)** The model scheme illustrating the flattening nucleus by actomyosin cytoskeleton opens the nuclear pore and faciliates the active transport of transcription factor YAP into nucleus. **(III)** The relationships between YAP nucleus/cytoplasm ratio ($R_{\text{NC}}$) and nuclear flattening ($\lambda_{N}$), as calculated by the model (line) and obtained by experiments on substrates with different ligand and stiffness (dots). A is adopted from reference [11]. B is adopted from reference [111].

In the first pathway of nuclear mechanotransduction, perinuclear actin generates active or passive forces that can induce changes in the condensation state of chromatin (Fig. 9A) [11]. Several studies have explored these mechanisms. One study by Damodaran et al. developed a 3D model that considered the passive compressive force inducing the mechanochemical feedbacks among focal adhesions, actomyosins and the nucleus [170]. The model revealed that compression on cells reduces actomyosin contractility by depolymerizing actin filaments, which further leads to the condensation of chromatin by reducing nuclear pressure [170]. Another study by Wei and colleagues constructed a finite element model to analyze the chromatin deformation and gene expression in response of different stress mode generated by ferromagnetic beads [171]. This model demonstrates that different force modes (magnetic twisting directions) result in varying cell stiffness while exhibiting similar levels of chromatin stretching and thus similar expressions of the DHFR gene [171]. These studies provide insights into the relationship between nuclear deformation and chromatin condensation. They highlight the role of actin-based forces in modulating the structure and function of the chromatin, ultimately influencing cellular behaviors and gene regulation.

In the second pathway of nuclear mechanotransduction, nuclear deformation plays a crucial role in controlling the transport of epigenetic and transcriptional factors [91,172]. Shenoy et al. have revealed through their 3D chemomechanical model that the tensile stresses generated by actin filaments can modify nuclear properties, which then determine the nucleocytoplasmic shuttling of epigenetic and transcription factors as [169].

(24) $$\frac{dN_{c}}{dt}=k_{\text{nc}}N_{n}-k_{\text{cn}}N_{c}$$

where $N_{c}$ and $N_{n}$ are cytoplasmic and nuclear concentration of epigenetic or transcription factors respectively, $k_{\text{nc}}$ and $k_{\text{cn}}$ are the rates of nucleus to cytoplasm transport and cytoplasm to nucleus transport respectively, and $k_{\text{nc}}$ depends on the contractility of actin filaments. Moreover, Elosegui-Artola et al. discovered a linear relationship between nucleus-to-cytoplasm (n/c) ratio of transcription factor YAP and the extent of nuclear flattening as [10]:

(25) $$R_{\text{NC}}=p\lambda_{N}+q$$

where $R_{\text{NC}}$ is YAP n/c ratio, $\lambda_{N}$ is the nuclear flattening (nuclear length/height), and p and q are fitting constant. Based on this relationship, our group found that the YAP associated “mechanical memory” can be explained by modeling the nucleus as a viscoelastic sphere (Fig. 9B, I) [111]:

(26) $$\varepsilon_{N}=\left(\frac{1}{E_{N}}+\frac{t}{\eta_{N}}\right)F_{\text{trac}}$$

where $\varepsilon_{N}$ is the nuclear strain and associated with $\lambda_{N}$ by a geometric relationship, $E_{N}$ and $\eta_{N}$ are effective elasticity and effective viscosity of nucleus respectively, t is the culturing time, $F_{\text{trac}}$ is the traction force generated on nucleus by actin filaments. The flattened nucleus opens its nuclear pores, facilitating the active transport of YAP into the nucleus (Fig. 9B, II) [111]. We examined the linear relationship between the n/c ratio of YAP and nuclear flattening among cells on different substrates (Fig. 9B, III) [111]. Such a phenomenon can be further explained by a continuum model of nuclear pores established by Liu et al. [173]. The model considers the nuclear pore complex as a basket structure that controls the transport of important macromolecules such as transcription factors. It reveals how stretching force on the nuclear membrane alters the conformation of the nuclear basket, enlarging the size of the nucleoplasmic ring [173].

## 5 Conclusions and outlook

Living cells possess the remarkable ability to adapt and respond to their surrounding microenvironments through dynamic interactions between their membranes, cytoskeleton, and nucleus. With the advancement of experimental techniques, more detailed mechanical phenomena associated with this active machinery have been uncovered. At the same time, the development of mechanical models provides us with a framework to understand the underlying mechanisms. In this review, we have provided an overview of how mechanical models have been employed to investigate the transmission of external forces on this mechanical integration. These models have effectively interpreted existing experimental observations and have highlighted future directions for cellular mechanobiology research. We are witnessing the evolutionary progression of these models towards exploring microscopic mechanisms, integrating complex mechanical and biochemical cues, and expanding their applications in various contexts.

While significant progress has been made in the field of cellular mechanobiology, there are still challenges that need to be addressed. One challenge is the difficulty in establishing a unified model that encompasses the holistic processes by which cells sense their microenvironment through the mechanical interactions of the membrane, cytoskeleton, and nucleus. With the discovery of more molecular elements involved in connecting, load bearing, and signaling within the cellular machinery, it becomes increasingly complex to include all these elements in a single model. Instead, we often have to analyze the roles of these elements on a case-by-case basis, continuously updating the models as new phenomena are discovered in mechanics-associated molecules.

Another challenge lies in the quantitative understanding of cellular mechanobiology. As an emerging interdisciplinary field, there are many unanswered quantitative questions that can only be addressed through the development of mechanical models. For example, we seek to understand how and to what extent a cell changes the expression and assembly of scaffold proteins in response to quantitative mechanical inputs such as stiffness and viscoelasticity of the extracellular matrix, concentration and pattern of ligands, and magnitude and frequency of cyclic loads or vibrational waves. We also aim to determine how these dynamic cellular structures influence cell mechanics, including adhesion strength, contraction force, and nuclear deformation. Additionally, we wonder how cell mechanics relate to cellular behaviors such as survival, proliferation, differentiation, and migration.

It is anticipated that answering these questions will require iterative advancements in both mechanical models and experimental techniques. The upgrading mechanical models will provide insights into these quantitative aspects of cellular mechanobiology, while novel experimental techniques will further validate and refine the models. Ultimately, answering these questions will deepen our understanding of cell biology and provide theoretical guidelines for addressing clinical problems.

## Author contributions

H.Z. and M.L. conceptualized the article. H.Z. and R.M. contributed to the text editions and figure designs. All authors contributed substantially to the discussion of the content and approved the final content.

## Ethical approval

This study does not contain any studies with human or animal subjects performed by any of the authors.

## Declaration of Generative AI and AI-assisted technologies in the writing process

During the preparation of this work the author(s) used ZJU/THU AI assistant in order to enhance the language quality. After using this tool/service, the author(s) reviewed and edited the content as needed and take(s) full responsibility for the content of the publication.

## Declaration of competing interest

The authors declare that they have no known competing financial interests or personal relationships that could have appeared to influence the work reported in this paper.
